# Perinatal outcomes among twin pregnancies with gestational diabetes mellitus: A nine-year retrospective cohort study

**DOI:** 10.3389/fpubh.2022.946186

**Published:** 2022-07-25

**Authors:** Dongxin Lin, Dazhi Fan, Pengsheng Li, Gengdong Chen, Jiaming Rao, Zixing Zhou, Huishan Zhang, Xin Luo, Huiting Ma, Jingping Feng, Demei Lu, Lijuan Wang, Shiyan Lan, Caihong Luo, Xiaoling Guo, Zhengping Liu

**Affiliations:** ^1^Foshan Institute of Fetal Medicine, Southern Medical University Affiliated Maternal and Child Health Hospital of Foshan, Foshan, China; ^2^Department of Obstetrics, Southern Medical University Affiliated Maternal and Child Health Hospital of Foshan, Foshan, China

**Keywords:** twin pregnancy, gestational diabetes mellitus, pregnancy outcome, neonatal outcome, perinatal outcome

## Abstract

**Objective:**

To compare the outcomes between gestational diabetes mellitus (GDM) vs. non-GDM twin gestations.

**Methods:**

A retrospective cohort study of 2,151 twin pregnancies was performed in a tertiary hospital in Foshan, China, 2012–2020. Pregnancy and neonatal outcomes were compared between women with vs. without GDM using 1:1 propensity score matching (PSM) and multivariable logistic models. For neonatal outcomes, generalized estimating equation (GEE) approach was used to address the intertwin correlation.

**Results:**

Of the 2,151 participants, 472 women (21.9%) were diagnosed with GDM. Women with GDM were older and more likely to be overweight or obese, and more likely have chronic hypertension, assisted pregnancies and dichorionic twins. In the PSM cohort of 942 pregnancies, there was no statistical difference when comparing GDM twin pregnancies and non-GDM in any of the perinatal outcomes, especially in terms of preterm birth (PTB) <37 weeks (*P* = 0.715), large for gestational age (LGA) (*P* = 0.521) and neonatal respiratory distress (NRDS) (*P* = 0.206). In the entire cohort, no significant adjusted ORs for these outcomes were obtained from logistic regression models adjusted for confounders (aOR for PTB < 37 weeks: 1.25, 95% CI: 0.98–1.58; aOR for LGA: 1.26, 95% CI: 0.88–1.82; and aOR for NRDS, 1.05, 95% CI: 0.68–1.64).

**Conclusion:**

Twin pregnancies with GDM and adequate prenatal care have comparable perinatal outcomes to those without.

## Introduction

Gestational diabetes mellitus (GDM) is one of the most common obstetric complications with an increasing prevalence worldwide, mainly due to the increasing prevalence of obesity in reproductive age, advanced maternal age, stricter diagnostic criteria and universal screening for GDM (1–3). This pregnancy complication is known to lead to short- and long-term adverse outcomes for both mothers and children, including hypertensive disorders of pregnancy (HDP), macrosomia, shoulder dystocia, cesarean section, neonatal respiratory morbidities, neonatal hypoglycemia, metabolic syndrome and cardiovascular disease (4–8).

Along with the increasing prevalence of GDM, the incidence of twin pregnancy has been increased in the last two decades because of the substantial improvement and expanded use of assisted reproductive technology (ART) (9, 10). Women with twin pregnancies have an increased risk of GDM compared to singletons (11, 12). It is recommended by the American College of Obstetrician and Gynecologist (ACOG) that the management of GDM twin gestations follows the same strategy for singleton gestations (13). However, it is uncertain whether the diagnostic criteria and glucose target for singleton is also appropriate for twin gestation, given that twin pregnancies are higher nutrition and energy demanded and have different clinical characteristics from singletons, such as more occurrence of prematurity and fetal growth retardation. Unfortunately, the current evidence on the outcomes among GDM twin pregnancies is limiting and conflicting. Some authors have reported that GDM twin pregnancies have similar outcomes with non-GDM ones (14, 15) whereas others have found GDM twin pregnancies with GDM have increased risks of adverse outcomes, including cesarean section (16–18), hypertensive complications (19–22), accelerated fetal growth (22–24) and neonatal respiratory morbidity (18). The conflicting findings may be due to several limitations including relatively small population, inadequate adjustment for confounders, and lack of addressing the intertwin correlation when analyzing neonatal outcomes (16, 18, 19).

In light of this, we aimed to compare the pregnancy and neonatal outcomes between GDM and non-GDM twin pregnancies.

## Methods

### Study design and population

This retrospective study was performed at a tertiary hospital in Foshan, China. The study population comprised women with twin pregnancies who gave birth beyond 26 weeks of gestation in the hospital from January 1^st^, 2012 to December 31^st^, 2020. In this study, pregnancies with congenital anomalies, twin-to-twin transfusion (TTTS), monoamniotic twins, unknown chorionicity, fetal loss or intrauterine death were excluded. To ensure the data completeness of maternal BMI, we excluded women who had their first prenatal visit after 14 weeks of gestation. Furthermore, based on the objective of the current study, those with pre-existing diabetes were excluded.

### Diagnosis of GDM

In the present study, GDM was diagnosed by an oral 75 g glucose tolerance test (OGTT) between 24 and 28 weeks of gestation if: fasting plasma glucose ≥ 5.1 mmol/L or 1-h plasma glucose ≥ 10.0 mmol/L or 2-h plasma glucose ≥ 8.5 mmol/L, according to the International Association of Diabetes and Pregnancy Study Groups (IADPSG) criteria (25).

### Baseline covariates

All electric medical records of eligible population were reviewed to collect relevant information on baseline characteristics, including demographic characteristics (maternal age, marital status, ethnicity and body mass index [BMI] at first trimester), obstetric characteristics (parity, mode of conception, chorionicity and fetal gender) and obstetric complications (chronic hypertension, pregnancy-induced hypertension [PIH], pre-eclampsia [PE], intrahepatic cholestasis of pregnancy [ICP] and hepatitis B). Maternal BMI was calculated by dividing weight (in kilograms) by height (in meters squared) and was categorized as underweight (BMI <18.5 kg/m^2^), normal weight (18.5–23.99 kg/m^2^), overweight (24–27.99 kg/m^2^) and obese (≥28 kg/m^2^) based on the standard of the Working Group on Obesity in China (26). Mode of conception included spontaneous and assisted reproductive technology (ART) conception. Chorionicity was assessed at the first sonographic examination before 14 weeks of gestations and confirmed by physical examination of the intertwin membranes after birth, if available. PIH was defined as a new development of a blood pressure of ≥140/90 mmHg after 20 weeks of gestation in the absence of proteinuria and PE was defined when a blood pressure of ≥140/90 mmHg and proteinuria of ≥300 mg/24 h were simultaneously found (27). ICP was diagnosed based on the presence of pruritus with elevated serum total bile acid (TBA) level (≥ 10 μmol/L).

### Outcomes of interest

Maternal outcomes of interest included preterm birth (PTB <37 weeks, <34 weeks and <32 weeks), cesarean section and premature rupture of membrane (PPROM). Neonatal outcomes of interest included low birth weight (<2,500 g), macrosomia (>4,000 g), small for gestational age (SGA), large for gestational age (LGA), neonatal respiratory distress syndrome (NRDS), neonatal asphyxia, ventilator support, neonatal jaundice, neonatal hypoglycemia, neonatal unit admission, bronchopulmonary dysplasia (BPD), necrotizing enterocolitis (NEC), hypoxic ischemic encephalopathy (HIE), intracranial hemorrhage (ICH), sepsis and neonatal death. SGA and LGA was defined when the birth weight was below or above the 10th percentile for gestational age and sex based on twin birthweight curves in Chinese twins, respectively (28, 29). Due to the low incidence of BPD, NEC, ICH, HIE, sepsis and neonatal death, we defined a composite outcome when any of these events occurred.

### Statistical analyses

All the statistical analyses were preformed using Stata, version 16.0. The baseline characteristics were reported as mean ± standard deviation (SD) or frequency with its percentage. Continuous variables with a Gaussian distribution were analyzed by independent sample *t* tests while categorical variables were analyzed by the Chi-square test or Fisher's exact test, where appropriate. Propensity score matching (PSM) was utilized to balance the baseline characteristics, including maternal age, ethnicity, BMI, nulliparity, mode of conception and chorionicity, between GDM vs. non-GDM pregnancies. We used 1:1 nearest-neighbor matching without replacement. A caliper width from 1 to 0 was tried until the absolute standardized mean difference (SMD) was below 0.1 for each covariate. A SMD of < 0.1 was regarded as a balance in the baseline characteristic between the study groups. After the PSM cohort was obtained, unadjusted logistic regressions were performed to evaluate the difference in maternal outcomes between GDM and non-GDM population. For neonatal outcomes, generalized estimating equation (GEE) approach was used to address the intertwin correlation and the models were further adjusted for gestational age at birth, which is a major factor of neonatal outcomes but not considered in PSM process. To ensure the robustness of the study finding, we also performed multivariable logistic regression on the entire cohort. Models for maternal outcomes were adjusted maternal age, BMI, nulliparity, mode of conception and chorionicity while the models for neonatal outcomes were further adjusted for gestational age at birth. Sensitivity analyses were performed by including women had missing information on maternal BMI (*n* = 874). The models were adjusted for maternal age, use of ART, nulliparity and chorionicity. The results were reported as odds ratio (ORs) and 95% confidence intervals (95% CIs). A two tailed *P*-value < 0.05 was considered statistical significance.

## Results

### Baseline characteristics of study population

**Figure 1:**
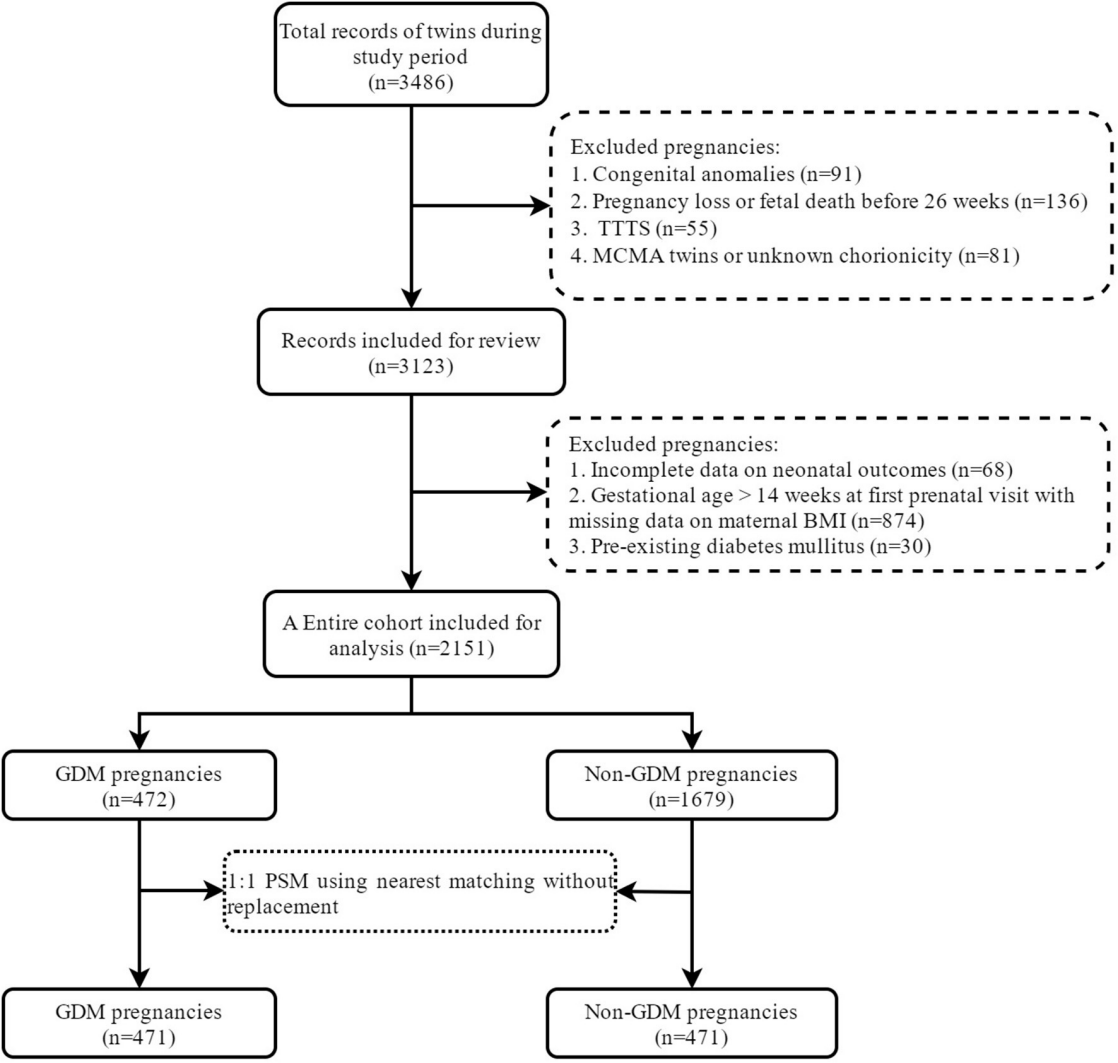
Selection of eligible participants.

A total of 2,151 twin pregnancies were included in the study, of which 472 were complicated with GDM with a prevalence of 21.9% (Figure 1). Table 1 shows the characteristics between GDM and non-GDM pregnancies. In general, women diagnosed with GDM were older (32.4 ± 4.3 vs. 30.7 ± 4.2, *P* < 0.001) and more likely to be overweight or obese than those without GDM (overweight: 22.3 vs. 15.5%; obesity: 9.3 vs. 3.5%). In addition, dichorionic twins (88.1 vs. 83.7%, *P* = 0.019) and use of ART (77.8 vs. 71.1%, *P* = 0.004) were more common in GDM pregnancies. The prevalence of chronic hypertension was also higher in GDM pregnant women (1.9 vs. 0.7%, *P* = 0.012). Other obstetric complications, including ICP, PIH, PE and hepatitis B, were similar between GDM and non-GDM groups. Among GDM women, only a small proportion (6.4%) used insulin during pregnancy. After PSM with a caliper width of 0.05, we obtained a total of 942 pregnancies (471 GDM pregnancies matched 1:1 to 471 non-GDM pregnancies) in the PSM cohort. The baseline characteristics of maternal age, ethnicity, nulliparity, mode of conception, chorionicity and maternal BMI were balanced between the two study groups (SMD <0.1).

**Table 1:** Baseline characteristics of the study population.

**Characteristics**	**Entire cohort**				**PSM cohort**			
	**Non-GDM (*****n** **=*** **1679)**	**GDM (*****n** **=*** **472)**	***P*** **-value**	**SMD**	**Non-GDM (*****n** **=*** **471)**	**GDM (*****n** **=*** **471)**	***P*** **-value**	**SMD**
Maternal age, year	30.7 ± 4.2	32.4 ± 4.3	<0.001	0.381	32.4 ± 4.4	32.3 ± 4.2	0.682	0.027
**Ethnicity**								
Han	1649 (98.2)	463 (98.1)	0.846	0.009	466 (98.9)	462 (98.1)	0.281	0.063
Others	30 (1.8)	9 (1.9)			5 (1.1)	9 (1.9)		
**Married**								
Yes	1620 (96.5)	448 (94.9)	0.118	-	461 (97.9)	447 (94.9)	0.014	-
No	59 (3.5)	24 (5.1)			10 (2.1)	24 (5.1)		
**Nulliparity**								
Yes	1165 (69.4)	324 (68.6)	0.758	0.016	313 (66.5)	323 (68.6)	0.487	0.046
No	514 (30.6)	148 (31.4)			158 (33.6)	148 (31.4)		
**Mode of conception**								
Spontaneous	485 (28.9)	105 (22.3)	0.004	0.153	102 (21.7)	105 (22.3)	0.813	0.015
ART	1194 (71.1)	367 (77.8)			369 (78.3)	366 (77.7)		
**Chorionicity**								
Dichorionic	406 (83.7)	416 (88.1)	0.019	0.127	420 (89.2)	415 (88.1)	0.608	0.031
Monochorionic	273 (16.3)	56 (11.9)			51 (10.8)	56 (11.9)		
**Maternal BMI**								
Underweight	278 (16.6)	36 (7.6)	<0.001	0.226	283 (60.1)	287 (60.9)	0.514	0.024
Normal weight	1083 (64.5)	287 (60.8)			47 (10)	36 (7.6)		
Overweight	260 (15.5)	105 (22.3)			105 (22.3)	104 (22.1)		
Obese	58 (3.5)	44 (9.3)			36 (7.6)	44 (9.3)		
**Use of insulin**								
Yes	-	30 (6.4)	-	-	-	30 (6.4)	-	-
No	-	442 (93.6)			-	442 (93.6)		
**Chronic hypertension**								
Yes	11 (0.7)	9 (1.9)	0.012	-	7 (1.5)	9 (1.9)	0.614	-
No	1668 (99.3)	463 (98.1)			464 (98.5)	462 (98.1)		
**Hepatitis B**								
Yes	112 (6.7)	40 (8.5)	0.177	-	44 (9.3)	40 (8.5)	0.647	-
No	1567 (93.3)	432 (91.5)			427 (90.7)	431 (91.5)		
**ICP**								
Yes	45 (2.7)	13 (2.8)	0.930	-	16 (3.4)	13 (2.8)	0.571	-
No	1634 (97.3)	459 (97.3)			455 (96.6)	458 (97.2)		
**PIH or PE**								
Yes	168 (10.0)	55 (11.7)	0.300	-	56 (11.9)	54 (11.5)	0.839	-
No	1511 (90.0)	417 (88.4)			415 (88.1)	417 (88.5)		
**Fetal sex**								
Male-male	558 (33.2)	152 (32.2)	0.375	-	159 (33.8)	151 (32.1)	0.855	-
Female-female	481 (28.7)	124 (26.3)			120 (25.5)	124 (26.3)		
Male-female	640 (38.1)	196 (41.5)			192 (40.8)	196 (41.6)		

PSM, propensity score matching; ART, assisted reproductive technology; BMI, body mass index; ICP, intrahepatic cholestasis of pregnancy; PIH, pregnancy-induced hypertension; PE, preeclampsia.

### Pregnancy outcomes between GDM and non-GDM twin pregnancies

Overall, the gestational age at delivery was similar between GDM pregnancies and non-GDM pregnancies (36.0 ± 1.8 vs. 35.9 ± 1.8, *P* = 0.121). After adjustment for confounders, the multivariable logistic regressions on the entire cohort showed no statistical difference in PTB between GDM and non-GDM pregnancies though the incidence of PTB <37 weeks was slightly higher in GDM pregnancies (OR, 1.25; 95% CI: 0.98–1.58; *P* = 0.082). The rates of cesarean section and PPROM were also similar between two groups. Based on the PSM cohort, there were no difference in the above-mentioned outcomes (Table 2).

**Table 2:** Maternal outcomes between GDM and non-GDM pregnancies.

	**Entire cohort**				**PSM cohort**			
**Outcomes**	**Non-GDM (*****n** **=*** **1679)**	**GDM (*****n** **=*** **472)**	**Adjusted OR** ^**a**^	***P*** **-value**	**Non-GDM (*****n** **=*** **471)**	**GDM (*****n** **=*** **471)**	**Unadjusted OR**	***P*** **-value**
PTB <37 weeks	1170 (69.7)	345 (73.1)	1.25 (0.98–1.58)	0.068	132 (28.0)	127 (27.0)	1.05 (0.79–1.40)	0.715
PTB <34 weeks	173 (10.3)	57 (12.1)	1.21 (0.88–1.68)	0.244	48 (10.2)	57 (12.1)	1.21 (0.81–1.82)	0.352
PTB <32 weeks	70 (4.2)	19 (4.0)	0.96 (0.56–1.62)	0.873	20 (4.3)	19 (4.0)	0.95 (0.50–1.80)	0.870
Cesarean section	1657 (98.7)	465 (98.7)	0.83 (0.35–1.99)	0.677	465 (98.7)	464 (98.5)	0.86 (0.29–2.56)	0.780
PPROM	218 (13.0)	65 (13.8)	1.09 (0.81–1.46)	0.575	59 (12.5)	71 (15.1)	1.24 (0.85–1.80)	0.258

PSM, propensity score matching; PTB, preterm birth; PROM, preterm premature rupture of membrane; ^a^, Models adjusted for maternal age, BMI, use of ART, nulliparity, and chorionicity.

### Neonatal outcomes between GDM and non-GDM twin pregnancies

In the entire cohort, there were no occurrence of macrosomia. The multivariable logistic models with GEE approach, adjusted for maternal age, BMI, use of ART, nulliparity, chorionicity, and gestational age at birth, showed no significant differences in the birthweight outcomes and other morbidities between GDM twins and non-GDM twins (Table 3). As the incidences of the severe morbidities (i.e, BPD, NEC, HIE, ICH, sepsis and neonatal death) were extremely low, the multivariable models were waived to calculate ORs for each morbidity. Regarding the composite outcome comprising any of these events, there was no difference between GDM and non-GDM (OR, 0.73; 95% CI, 0.38–1.38). Based on the PSM cohort, the models were adjusted for gestational age at delivery. No significant differences were found in abovementioned outcomes between twin newborns with and without maternal GDM.

**Table 3:** Neonatal outcomes between GDM and non-GDM pregnancies.

	**Entire cohort**				**PSM cohort**			
**Outcomes**	**Non-GDM (*****n** **=*** **3358)**	**GDM (*****n** **=*** **944)**	**Adjusted OR** ^**a**^	***P*** **-value**	**Non-GDM (*****n** **=*** **942)**	**GDM (*****n** **=*** **942)**	**Adjusted OR** ^**b**^	***P*** **-value**
Low birth weight (<2,500 g)	2106 (62.7)	595 (63.0)	0.98 (0.81–1.18)	0.831	565 (60.0)	594 (63.1)	1.1 (0.87–1.39)	0.425
Macrosomia (>4,000 g)	0 (0)	0 (0)	-	-			-	-
Small for gestational age	228 (6.8)	66 (7.0)	1.07 (0.79–1.43)	0.673	58 (6.2)	65 (6.9)	1.12 (0.77–1.64)	0.562
Large for gestational age	152 (4.5)	56 (5.9)	1.26 (0.88–1.82)	0.205	49 (5.2)	56 (5.9)	1.16 (0.74–1.82)	0.521
NRDS	264 (7.9)	77 (8.2)	1.05 (0.68–1.64)	0.816	59 (6.3)	77 (8.2)	1.47 (0.81–2.69)	0.206
Neonatal asphyxia	60 (1.8)	24 (2.5)	1.40 (0.78–2.52)	0.264	16 (1.7)	24 (2.6)	1.33 (0.60–2.94)	0.478
Ventilator support	226 (6.7)	61 (6.5)	0.85 (0.55–1.31)	0.456	56 (5.9)	61 (6.5)	1.00 (0.58–1.74)	0.987
Neonatal jaundice	950 (28.3)	286 (30.3)	0.97 (0.77–1.23)	0.822	260 (27.6)	285 (30.3)	1.09 (0.83–1.42)	0.548
Neonatal hypoglycemia	114 (3.4)	41 (4.3)	1.35 (0.89–2.04)	0.158	30 (3.2)	41 (4.4)	1.35 (0.79–2.29)	0.269
Neonatal unit admission	1324 (39.4)	397 (42.1)	0.99 (0.80–1.24)	0.959	369 (39.2)	395 (42.0)	1.07 (0.81–1.41)	0.626
BPD	49 (1.5)	8 (0.9)	-	-	8 (0.9)	8 (0.9)	-	-
NEC	28 (0.8)	3 (0.3)	-	-	5 (0.5)	3 (0.3)	-	-
HIE	12 (0.4)	3 (0.3)	-	-	1 (0.1)	3 (0.3)	-	-
ICH	21 (0.6)	3 (0.3)	-	-	2 (0.2)	3 (0.3)	-	-
Sepsis	40 (1.2)	8 (0.9)	-	-	8 (0.9)	8 (0.9)	-	-
Neonatal death	13 (0.4)	3 (0.3)	-	-	2 (0.2)	3 (0.3)	-	-
Severe composite outcome	78 (2.3)	19 (2.0)	0.73 (0.38–1.38)	0.333	19 (2.0)	21 (2.2)	0.86 (0.41–1.81)	0.687

PSM, propensity score matching; NRDS, neonatal respiratory distress syndrome; BPD, bronchopulmonary dysplasia; HIE, hypoxic ischemic encephalopathy; ICH, intracranial hemorrhage; ^a^, GEE models adjusted for maternal age, BMI, use of ART, nulliparity, and chorionicity and gestational age at delivery; ^b^, GEE models adjusted for gestational age at delivery.

### Sensitivity analyses

The baseline characteristics were compared between those with missing information on maternal BMI vs. those without. Maternal age, nulliparity, mode of conception, chorionicity, chronic hypertension, hepatitis B and PIH or PE were found to be different between these population (Supplementary Table S1). After including those with first prenatal visit after 14 weeks of gestation, an increased aOR for PTB <34 weeks (aOR, 1.40; 95% CI: 1.08–1.81) was found (Supplementary Table S2). No other significant aORs were found in the sensitivity analysis including women who had their first visit after 14 weeks of gestational age (Supplementary Table S3).

## Discussion

In the current study, we compared the perinatal outcomes between twin pregnancies with vs. without GDM. Overall, the prevalence of GDM in twin pregnancies was 21.9%. Both of the PSM and the entire cohort revealed that the maternal and neonatal outcomes were comparable between GDM and non-GDM twin pregnancies.

The prevalence of GDM depends on the use of screening methods, population characteristics and diagnostic criteria. Based on the IADPSG criteria, we obtained a prevalence similar to that reported in another Chinese population with twin pregnancies (20.4%) (30). In line with previous reports (14, 15, 23, 31), women with GDM were of advanced age and higher BMI. Conversely, our finding, that the incidence of PIH or PE between GDM and non-GDM women was similar, was conflicting with previous studies, which reported a relationship between GDM and HDP in both singleton and twin pregnancies (15, 21, 32). We also found that GDM pregnancies were more likely to be assisted conception, which might be affected by maternal age and BMI because women with advanced age or obesity are likely to be in need of fertility treatment (33).

Regarding the maternal outcomes, we did not observe an increased risk of preterm birth among GDM twin pregnancies, whether in the PSM or the entire cohort. This finding supported previous findings (14, 17, 20) but that of Hiersch et al. (23). The lack of association could be partially explained by the similar incidence of PIH and PE and PPROM in both groups. Also, since this study was performed at a tertiary hospital, pregnancies included in this study were more likely to be high-risk, resulting in iatrogenic preterm delivery, even in the non-GDM group. Not unexpectedly, different from singleton pregnancies, the rate of cesarean section was much higher among twin gestations. Apart from common growth retardation and breech presentation among twins, the high rate of cesarean section in current study population could be attributed to maternal inclination to deliver babies by cesarean section ahead of expected date since vaginal delivery is thought to be risky. In this regard, the observed difference in cesarean section rate between GDM and non-GDM twin pregnancies was blunted.

Accumulated evidence that GDM pregnancies are at risk of accelerated fetal growth are well established in singletons (34–37). For twin pregnancies, whereas, the situation might be different. In the current study, no macrosomia was found in both GDM and non-GDM pregnancies, mainly due to the generally earlier delivery and slower growth among twin pregnancies compared with singleton pregnancies. We did not observe difference in neither LGA nor SGA between GDM and non-GDM pregnancies, which was consistent with previous findings (14, 17, 19, 20). Ashwal et al. (38) recently reported that the longitudinal fetal growth was different between GDM and non-GDM singleton fetuses but comparable between GDM and non-GDM twin fetuses. In this regard, the fetal overgrowth in GDM twin pregnancies might be counteracted by the inherent nature of decreased fetal growth velocity in the third trimester and increased predisposition to fetal growth restriction. Similarly, there was no relationship between neonatal respiratory morbidities and GDM among our study population, consistent with previous reports (17, 20, 23). Maternal diabetes, including GDM and pre-existing diabetes, is reported to be associated with delayed fetal lung maturation, which causes neonatal respiratory morbidities (39–41). This evidence came from singleton pregnancies. Nevertheless, twins showed more rapid fetal lung development than singletons, as reported by Tsuda et al. (42). Further evidence is necessary to clarify different roles of GDM in the lung maturation between GDM twins and singletons.

Luo et al. (24) speculated that the prolong elevated glucose level might play a protective role in multiple pregnancies, which are higher energy and nutrient demanded. The finding of similar neonatal outcomes between GDM and non-GDM twin pregnancies could suggest different clinical implications of GDM for twins and singletons. It also raised our rethinking, whether the diagnostic criteria and prenatal management for GDM should be individualized for twin pregnancies.

The main strength of the present study was the relatively large study population. Furthermore, in the analysis, we adopted PSM method and the results were similar to those obtained from the multivariable models on the entire cohort, which increased the robustness of our finding. Several limitations should be aware of when interpreting the current results, however. First, this study was based on a single center in China, which would limit the generalization of our results to other populations. Furthermore, due to the retrospective design, this database did not include information on oral glucose tolerance test (OGTT) and degree of glycemic control during gestation, which limited the interpretation of the findings. A previous study reported that accelerated fetal growth was found in medically-treated GDM but not diet-treated GDM twin pregnancies, compared with non-GDM pregnancies (38). In the current study, only a small proportion of GDM women used insulin, which may indicate relatively good glycemic control in our study population. Other information, such as maternal smoking status, gestational weight gain, Apgar score, was not available or incomplete in our database. These residual confounding cannot be rule out. In this study, we used the measured weight at the first prenatal visit before 14 weeks to calculate the maternal BMI, which may introduce bias, despite its satisfying correlation with pre-pregnancy weight (32). At last, selection bias could be caused by excluding pregnant women with their first visit after 14 weeks of gestations to ensure information completeness of maternal BMI. The main sample who attended their prenatal visit before 14 weeks of gestations would have higher adequacy of prenatal care or better adherence to prenatal care. Our sensitivity analyses revealed an increased risk of PTB <34 weeks in GDM pregnancies after including those with their first visit after 14 weeks of gestations. This result might also stress the important role of timing of initial antenatal care in GDM twin pregnancies.

In conclusion, we found that, GDM twin pregnancies with adequate prenatal care have similar outcomes to non-GDM pregnancies.

## Data availability statement

The raw data supporting the conclusions of this article will be made available by the authors, without undue reservation.

## Ethics statement

The studies involving human participants were reviewed and approved by Southern Medical University Affiliated Maternal and Child Health Hospital of Foshan. The Ethics Committee waived the requirement of written informed consent for participation.

## Author contributions

DLin and ZL: concept and design. HZ, JR, ZZ, JF, DLu, LW, SL, and CL: acquisition, analysis, and interpretation of data. DLin, GC, and PL: statistical analysis. DLin and DF: drafting of the manuscript. XL, XG, HM, and ZL: critical revision of the manuscript. All authors contributed to the article and approved the submitted version.

## Conflict of interest

The authors declare that the research was conducted in the absence of any commercial or financial relationships that could be construed as a potential conflict of interest.

## Publisher's note

All claims expressed in this article are solely those of the authors and do not necessarily represent those of their affiliated organizations, or those of the publisher, the editors and the reviewers. Any product that may be evaluated in this article, or claim that may be made by its manufacturer, is not guaranteed or endorsed by the publisher.

## References

[1] Griffin ME Coffey M Johnson H Scanlon P Foley M Stronge J. Universal vs. risk factor-based screening for gestational diabetes mellitus: detection rates, gestation at diagnosis and outcome. Diabetic Med J Br Diabetic Assoc. (2000) 17:26–32. 10.1046/j.1464-5491.2000.00214.x 10691156

[2] Ferrara A. Increasing prevalence of gestational diabetes mellitus: a public health perspective. Diabetes Care. (2007) 30(Suppl 2):S141–6. 10.2337/dc07-s206 17596462

[3] Saeedi M Cao Y Fadl H Gustafson H Simmons D. Increasing prevalence of gestational diabetes mellitus when implementing the Iadpsg criteria: a systematic review and meta-analysis. Diabetes Res Clin Pract. (2021) 172:108642. 10.1016/j.diabres.2020.108642 33359574

[4] Ramezani Tehrani F Naz MSG Yarandi RB Behboudi-Gandevani S. The impact of diagnostic criteria for gestational diabetes mellitus on adverse maternal outcomes: a systematic review and meta-analysis. J Clin Med. (2021) 10:666. 10.3390/jcm10040666 33572314 PMC7916110

[5] Hosseini E Janghorbani M. Systematic review and meta-analysis of diagnosing gestational diabetes mellitus with one-step or two-step approaches and associations with adverse pregnancy outcomes. Int J Gynaecol Obstet. (2018) 143:137–44. 10.1002/ijgo.12644 30101526

[6] Miao Z Wu H Ren L Bu N Jiang L Yang H. Long-term postpartum outcomes of insulin resistance and B-cell function in women with previous gestational diabetes mellitus. Int J Endocrinol. (2020) 2020:7417356. 10.1155/2020/7417356 32184821 PMC7061142

[7] Nijs H Benhalima K. Gestational diabetes mellitus and the long-term risk for glucose intolerance and overweight in the offspring: a narrative review. J Clin Med. (2020) 9:599. 10.3390/jcm9020599 32098435 PMC7074239

[8] Leybovitz-Haleluya N Wainstock T Landau D Sheiner E. Maternal gestational diabetes mellitus and the risk of subsequent pediatric cardiovascular diseases of the offspring: a population-based cohort study with up to 18 years of follow up. Acta Diabetol. (2018) 55:1037–42. 10.1007/s00592-018-1176-1 29936651

[9] Smits J Monden C. Twinning across the developing world. PLoS ONE. (2011) 6:e25239. 10.1371/journal.pone.0025239 21980404 PMC3182188

[10] Adashi EY Gutman R. Delayed childbearing as a growing, previously unrecognized contributor to the national plural birth excess. Obstet Gynecol. (2018) 132:999–1006. 10.1097/AOG.0000000000002853 30204699

[11] Schwartz DB Daoud Y Zazula P Goyert G Bronsteen R Wright D. Gestational diabetes mellitus: metabolic and blood glucose parameters in singleton versus twin pregnancies. Am J Obstet Gynecol. (1999) 181:912–4. 10.1016/s0002-9378(99)70324-8 10521752

[12] Hiersch L Berger H Okby R Ray JG Geary M McDonald SD. Incidence and risk factors for gestational diabetes mellitus in twin versus singleton pregnancies. Arch Gynecol Obstet. (2018) 298:579–87. 10.1007/s00404-018-4847-9 29971559

[13] American College of Obstetricians and Gynecologists. Multifetal gestations: twin, triplet, and higher-order multifetal pregnancies: acog practice bulletin, number 231. Obstet Gynecol. (2021) 137:e145–e62. 10.1097/AOG.0000000000004397 34011891

[14] Hung TH Hsieh TT Shaw SW Kok Seong C Chen SF. Risk factors and adverse maternal and perinatal outcomes for women with dichorionic twin pregnancies complicated by gestational diabetes mellitus: a retrospective cross-sectional study. J Diabetes Investig. (2021) 12:1083–91. 10.1111/jdi.13441 33064935 PMC8169347

[15] Okby R Weintraub AY Sergienko R Eyal S. Gestational diabetes mellitus in twin pregnancies is not associated with adverse perinatal outcomes. Arch Gynecol Obstet. (2014) 290:649–54. 10.1007/s00404-014-3272-y 24823995

[16] Moses RG Webb AJ Lucas EM Davis WS. Twin pregnancy outcomes for women with gestational diabetes mellitus compared with glucose tolerant women. Aust N Z J Obstet Gynaecol. (2003) 43:38–40. 10.1046/j.0004-8666.2003.00015.x 12755345

[17] Alkaabi J Almazrouei R Zoubeidi T Alkaabi FM Alkendi FR Almiri AE. Burden, associated risk factors and adverse outcomes of gestational diabetes mellitus in twin pregnancies in Al Ain, Uae. BMC Pregn Childbirth. (2020) 20:612. 10.1186/s12884-020-03289-w 33046000 PMC7552445

[18] Rauh-Hain JA Rana S Tamez H Wang A Cohen B Cohen A. Risk for developing gestational diabetes in women with twin pregnancies. J Maternal-Fetal Neonatal Med. (2009) 22:293–9. 10.1080/14767050802663194 19340713

[19] Guillén MA Herranz L Barquiel B Hillman N Burgos MA Pallardo LF. Influence of gestational diabetes mellitus on neonatal weight outcome in twin pregnancies. Diabetic Med J Br Diabetic Assoc. (2014) 31:1651–6. 10.1111/dme.12523 24925592

[20] González González NL Goya M Bellart J Lopez J Sancho MA Mozas J. Obstetric and perinatal outcome in women with twin pregnancy and gestational diabetes. J Maternal-Fetal Neonatal Med. (2012) 25:1084–9. 10.3109/14767058.2011.622009 21919552

[21] Dinham GK Henry A Lowe SA Nassar N Lui K Spear V. Twin pregnancies complicated by gestational diabetes mellitus: a single centre cohort study. Diabetic Med J Br Diabetic Assoc. (2016) 33:1659–67. 10.1111/dme.13076 26802478

[22] Mei Y Yu J Wen L Fan X Zhao Y Li J. Perinatal outcomes and offspring growth profiles in twin pregnancies complicated by gestational diabetes mellitus: a longitudinal cohort study. Diabetes Res Clin Pract. (2021) 171:108623. 10.1016/j.diabres.2020.108623 33316314

[23] Hiersch L Berger H Okby R Ray JG Geary M McDonald SD. Gestational diabetes mellitus is associated with adverse outcomes in twin pregnancies. Am J Obst Gynecol. (2019) 220:102.e1–.e8. 10.1016/j.ajog.2018.10.027 30595142

[24] Luo ZC Simonet F Wei SQ Xu H Rey E Fraser WD. Diabetes in pregnancy may differentially affect neonatal outcomes for twins and singletons. Diabetic Med J Br Diabetic Assoc. (2011) 28:1068–73. 10.1111/j.1464-5491.2011.03366.x 21679236

[25] Metzger BE Gabbe SG Persson B Buchanan TA Catalano PA Damm P. International association of diabetes and pregnancy study groups recommendations on the diagnosis and classification of hyperglycemia in pregnancy. Diabetes Care. (2010) 33:676–82. 10.2337/dc09-1848 20190296 PMC2827530

[26] Wang Y Mi J Shan XY Wang QJ Ge KY. Is China facing an obesity epidemic and the consequences? the trends in obesity and chronic disease in China. Int J Obesity. (2007) 31:177–88. 10.1038/sj.ijo.0803354 16652128

[27] American College of Obstetricians and Gynecologists. Hypertension in Pregnancy. Report of the American College of obstetricians and gynecologists' task force on hypertension in pregnancy. Obst Gynecol. (2013) 122:1122–31. 10.1097/01.AOG.0000437382.03963.88 24150027

[28] Zhang B Cao Z Zhang Y Yao C Xiong C Zhang Y. Birthweight percentiles for twin birth neonates by gestational age in China. Sci Rep. (2016) 6:31290. 10.1038/srep31290 27506479 PMC4978964

[29] Dai L Deng C Li Y Yi L Li X Mu Y. Population-based birth weight reference percentiles for Chinese twins. Ann Med. (2017) 49:470–8. 10.1080/07853890.2017.1294258 28276868

[30] Liu X Chen Y Zhou Q Shi H Cheng WW. Utilization of international association of diabetes and pregnancy study groups criteria vs. a two-step approach to screening for gestational diabetes mellitus in Chinese women with twin pregnancies. Diabetic Med J Br Diabetic Assoc. (2015) 32:367–73. 10.1111/dme.12636 25407306

[31] Wen L Ge H Qiao J Zhang L Chen X Kilby MD. Maternal dietary patterns and risk of gestational diabetes mellitus in twin pregnancies: a longitudinal twin pregnancies birth cohort study. Nutr J. (2020) 19:13. 10.1186/s12937-020-00529-9 32039726 PMC7008526

[32] Mdoe MB Kibusi SM Munyogwa MJ Ernest AI. Prevalence and predictors of gestational diabetes mellitus among pregnant women attending antenatal clinic in dodoma region, tanzania: an analytical cross-sectional study. BMJ Nutr Prevent Health. (2021) 4:69–79. 10.1136/bmjnph-2020-000149 34308114 PMC8258095

[33] Lin D Li P Fan D Chen G Wu S Ye S. Association between Ivf/Icsi treatment and preterm birth and major perinatal outcomes among dichorionic-diamnionic twin pregnancies: a seven-year retrospective cohort study. Acta Obstet Gynecol Scand. (2021) 100:162–9. 10.1111/aogs.13981 32865233

[34] Yang GR Dye TD Li D. Effects of pre-gestational diabetes mellitus and gestational diabetes mellitus on macrosomia and birth defects in upstate New York. Diabetes Res Clin Pract. (2019) 155:107811. 10.1016/j.diabres.2019.107811 31401151 PMC8783133

[35] He XJ Qin FY Hu CL Zhu M Tian CQ Li L. is gestational diabetes mellitus an independent risk factor for macrosomia: a meta-analysis? Arch Gynecol Obstet. (2015) 291:729–35. 10.1007/s00404-014-3545-5 25388922

[36] Aviram A Guy L Ashwal E Hiersch L Yogev Y Hadar E. Pregnancy outcome in pregnancies complicated with gestational diabetes mellitus and late preterm birth. Diabetes Res Clin Pract. (2016) 113:198–203. 10.1016/j.diabres.2015.12.018 26810272

[37] Metzger BE Lowe LP Dyer AR Trimble ER Chaovarindr U Coustan DR. Hyperglycemia and adverse pregnancy outcomes. N Engl J Med. (2008) 358:1991–2002. 10.1056/NEJMoa0707943 18463375

[38] Ashwal E Berger H Hiersch L Yoon EW Zaltz A Shah B. Gestational diabetes and fetal growth in twin compared with singleton pregnancies. Am J Obstet Gynecol. (2021) 225:420-e1. 10.1016/j.ajog.2021.04.225 33872592

[39] Robert MF Neff RK Hubbell JP Taeusch HW Avery ME. Association between maternal diabetes and the respiratory-distress syndrome in the newborn. N Engl J Med. (1976) 294:357–60. 10.1056/NEJM197602122940702 1246288

[40] Vignoles P Gire C Mancini J Bretelle F Boubli L Janky E. Gestational diabetes: a strong independent risk factor for severe neonatal respiratory failure after 34 weeks. Arch Gynecol Obstet. (2011) 284:1099–104. 10.1007/s00404-010-1810-9 21170541

[41] Mortier I Blanc J Tosello B Gire C Bretelle F Carcopino X. Is Gestational diabetes an independent risk factor of neonatal severe respiratory distress syndrome after 34 weeks of gestation? A prospective study. Arch Gynecol Obstet. (2017) 296:1071–7. 10.1007/s00404-017-4505-7 28948345

[42] Tsuda H Kotani T Nakano T Imai K Ushida T Hirakawa A. The rate of neonatal respiratory distress syndrome/transient tachypnea in the newborn and the amniotic lamellar body count in twin pregnancies compared with singleton pregnancies. Clin Chim Acta. (2018) 484:293–7. 10.1016/j.cca.2018.06.015 29894780

