# Supplementary material for: Perinatal outcomes among twin pregnancies with gestational diabetes mellitus: A nine-year retrospective cohort study
Source: Front Public Health. 2022 Jul 25;10:946186. doi: 10.3389/fpubh.2022.946186 (PMC9358001; doi:10.3389/fpubh.2022.946186)
Supplement: Supplementary file 1 [file Table_1.pdf]

Table S1. Comparison of baseline characteristics between women who had their first prenatal visit before versus after 14 weeks of gestations.

Characteristics	Before 14 weeks (n=2151)	After 14 weeks (n=874)	*P*-value
Maternal age, year	31.1±4.3	30.1±5.4	<0.001
Ethnicity			
Han	39 (1.8)	22 (2.5)	0.212
Others	2112 (98.2)	852 (97.5)	
Married			
Yes	2068 (96.1)	827 (94.6)	0.062
No	83 (3.9)	47 (5.4)	
Nulliparity			
Yes	1489 (69.2)	549 (62.8)	0.001
No	662 (30.8)	325 (37.2)	
Mode of conception			
Spontaneous	590 (27.4)	456 (52.2)	<0.001
ART	1561 (72.6)	418 (47.8)	
Chorionicity			
Dichorionic	1822 (84.7)	661 (75.6)	<0.001
Monochorionic	329 (15.3)	213 (24.4)	
Maternal BMI			
Underweight	-	-	-
Normal weight	-	-	
Overweight	-	-	
Obese	-	-	
Use of insulin			
Yes	30 (6.4)	6 (4.2)	0.343
No	442 (93.6)	136 (95.8)	
Chronic hypertension			
Yes	20 (0.9)	1 (0.1)	0.013
No	2131 (99.1)	873 (99.9)	
Hepatitis B			
Yes	152 (7.1)	83 (9.5)	0.024
No	1999 (92.9)	791 (90.5)	
ICP			
Yes	58 (2.7)	22 (2.5)	0.781
No	2093 (97.3)	852 (97.5)	
PIH or PE			
Yes	223 (10.4)	114 (13.0)	0.034
No	1928 (89.6)	760 (87.0)	
Fetal sex			
Male-male	710 (33.0)	343 (39.2)	<0.001
Female-female	605 (28.1)	264 (30.2)	
Male-female	836 (38.9)	267 (30.6)	

Table S2. Maternal outcomes between GDM and non-GDM pregnancies (including women who had their first visit after 14 weeks of gestational age).

Outcomes	Non-GDM (n=2411)	GDM (n=614)	Adjusted OR[a]	*P*-value
PTB <37 weeks	1728 (71.7)	452 (73.6)	1.14 (0.93-1.40)	0.212
PTB <34 weeks	303 (12.6)	94 (15.3)	1.40 (1.08-1.81)	0.010
PTB <32 weeks	139 (5.8)	37 (6.0)	1.15 (0.79-1.69)	0.460
Caesarean section	2361 (97.9)	601 (97.9)	0.78 (0.41-1.46)	0.435
PPROM	372 (15.4)	99 (16.1)	1.11 (0.87-1.42)	0.409

[a], Models adjusted for maternal age, use of ART, nulliparity and chorionicity.

Table S3. Neonatal outcomes between GDM and non-GDM pregnancies (including women who had their first visit after 14 weeks of gestational age)

Outcomes	Non-GDM (n=4822)	GDM (n=1228)	Adjusted OR[a]	*P*-value
Low birth weight (<2500 g)	3108 (64.5)	777 (63.3)	1.03 (0.88-1.21)	0.683
Macrosomia (>4000 g)	0 (0)	0 (0)	-	-
Small for gestational age	367 (7.6)	88 (7.2)	0.98 (0.76-1.27)	0.894
Large for gestational age	209 (4.3)	74 (6.0)	1.35 (1.00-1.84)	0.054
NRDS	447 (9.3)	127 (10.3)	1.26 (0.95-1.67)	0.116
Neonatal asphyxia	126 (2.6)	32 (2.6)	1.06 (0.65-1.75)	0.808
Ventilator support	393 (8.2)	110 (9)	1.20 (0.90-1.61)	0.212
Neonatal jaundice	1449 (30.1)	380 (30.9)	1.12 (0.94-1.33)	0.209
Neonatal hypoglycemia	189 (3.9)	49 (4.0)	1.06 (0.73-1.53)	0.758
Neonatal unit admission	1987 (41.3)	526 (42.9)	1.13 (0.95-1.33)	0.158
BPD	68 (1.4)	18 (1.5)	-	-
NEC	39 (0.8)	3 (0.2)	-	-
HIE	15 (0.3)	3 (0.2)	-	-
ICH	27 (0.6)	3 (0.2)	-	-
Sepsis	65 (1.4)	10 (0.8)	-	-
Neonatal death	26 (0.5)	3 (0.2)	-	-
Severe composite outcome	141 (2.9)	33 (2.7)	0.95 (0.59-1.52)	0.829

NRDS, neonatal respiratory distress syndrome; BPD, bronchopulmonary dysplasia; HIE, hypoxic ischemic encephalopathy; ICH, intracranial hemorrhage; [a], GEE models adjusted for maternal age, BMI, use of ART, nulliparity, chorionicity and gestational age at delivery.
